# Utilization and associated factors of long-lasting insecticide-treated nets among households in Gondar Zuria district, Northwest Ethiopia: a mixed methods study, 2025

**DOI:** 10.1186/s12936-026-05839-7

**Published:** 2026-03-04

**Authors:** Desalegn Tesfaye Begashaw, Meron Asmamaw Alemayehu, Yaregal Animut, Shegaw Marie

**Affiliations:** 1 Field Epidemiology and Laboratory Training Program, University of Gondar, Gondar, Ethiopia; 2 Department of Epidemiology and Biostatistics, Institute of Public Health, College of Medicine and Health Science, University of Gondar, Gondar, Ethiopia; 3 Department of Health Informatics, Institute of Public Health, College of Medicine and Health Science, University of Gondar, Gondar, Ethiopia; 4 Center for Digital Health and Implementation Science, College of Medicine and Health Science, University of Gondar, Gondar, Ethiopia

**Keywords:** Ethiopia, LLINs, Utilization,mixed method, Malaria

## Abstract

**Background:**

Malaria is a major public health problem in Ethiopia, and long-lasting insecticide-treated nets (LLINs) are one of the primary preventive strategies used to control it. Gondar Zuria District, Central Gondar Zone's highest malaria-reporting district, reached 100% LLIN coverage in 2023. However, malaria cases remain high, suggesting gaps in LLIN utilization. Therefore, this study aimed to assess LLIN usage and determinants in Gondar Zuria District.

**Methods:**

Community-based mixed-methods study was conducted from February 1 to 28, 2025 among 700 participants selected through multistage sampling. Quantitative data was collected via interviewer-administered questionnaires and entered into EpiData version 4.6.0.2 and exported to SPSS version 25 for analysis. A binary logistic regression model was fitted, and statistical significance was determined using 95% confidence intervals (CI) and a p-value ≤ 0.05. Qualitative data were collected through purposively sampled interviews (n = 7) and key informant interviews (n = 3) and analyzed thematically.

**Results:**

The prevalence of ITN utilization was 55.6% (95% CI: 51.9%–59.3%) and was significantly associated with perceived severity (AOR = 1.74; 95% CI: 1.27–2.39), LLINs-to-family size ratio (AOR = 2.09; 95% CI: 1.51–2.91), and occupation specifically, being a government employee (AOR = 3.45; 95% CI: 1.57–7.58). The mixed methods design therefore provided a more comprehensive understanding by revealing behavioral and contextual factors that were not captured through quantitative data alone. Qualitative findings complemented the quantitative results by explaining why LLIN utilization remained low, highlighting misconceptions about net safety, doubts about effectiveness, discomfort due to heat, and structural barriers such as lack of space for hanging nets.

**Conclusion:**

LLIN utilization in Gondar Zuria District fell below the national target (100%). Misconceptions and misinformation contribute to irregular use and reduced trust. Beyond distribution and health education, community-based behavior changes strategies such as household follow-ups by health workers and integrating LLIN promotion into local forums alongside routine net condition monitoring are essential to improve consistent use and advance malaria elimination efforts.

## Introduction

Malaria continues to pose a major public health challenge in sub-Saharan Africa, with Ethiopia among the countries bearing a high burden of the disease. It is a life-threatening illness caused by *Plasmodium* parasites transmitted through the bites of infected female *Anopheles* mosquitoes [1]. Although it is preventable and treatable, malaria remains endemic in many regions of Ethiopia, particularly in lowland and riverine areas. The World Health Organization (WHO) has long recommended vector control especially through insecticide-treated nets (ITNs) and indoor residual spraying (IRS) as a cornerstone of malaria prevention [2]. Among these, Long-Lasting Insecticide-Treated Nets (LLINs) have been instrumental in reducing malaria morbidity and mortality when properly utilized [3].

Since the year 2000, widespread LLIN distribution campaigns and improved malaria case management have led to significant reductions in malaria incidence globally, with an estimated 50% decline in malaria cases in several endemic countries [2, 4]. In sub-Saharan Africa, LLINs have contributed to saving millions of lives, especially among children under five, where community-wide trials showed a 20% reduction in all-cause mortality [5]. The WHO now recommends universal LLIN coverage, ensuring that all individuals in malaria-prone areas not just pregnant women and young children have access to nets [4].

In Ethiopia, the National Malaria Elimination Strategic Plan sets ambitious goals to eliminate malaria from low-transmission districts by 2025 and from the entire country by 2030 [6]. To this end, large-scale LLIN distribution campaigns have been launched every three years, targeting one net per two people. As a result, LLIN ownership has increased significantly. In 2023, Gondar Zuria District reported 100% household coverage [7]. However, a critical issue remains. Ownership of LLINs does not equate to proper utilization. Malaria incidence remains high in Gondar Zuria, suggesting that while distribution goals have been met, behavioral and contextual factors may be undermining the effectiveness of this intervention.

The discrepancy between ownership and utilization of LLINs has been documented in several studies. Nationally, the LLIN utilization rate in Ethiopia stands at 56.3% in 2023, while the Amhara Region, where Gondar Zuria is located, reports slightly higher rates at 63% [8]. In some districts, utilization rates fall well below 50% despite near-universal ownership. For example, a study in the Southwest Ethiopian Peoples' Region found that although 89% of households owned LLINs, only 20.4% used them properly [9]. However, most previous studies have focused mainly on measuring ownership and general utilization levels and have not fully explored the reasons for the persistent low use of LLINs despite widespread distribution. This gap highlights the need for further investigation into why universal LLIN coverage has not translated into improved utilization in high-burden districts like Gondar Zuria.

Numerous studies in Ethiopia and across Africa have identified factors influencing LLIN use. Sociodemographic characteristics such as sex, age, marital status, education, and occupation have consistently shown associations with LLIN utilization. For instance, older household heads and those with higher education are more likely to use LLINs [10, 11]. A cross-sectional study in Benishangul Gumuz revealed that households with fewer members (≤ 4) were twice as likely to utilize LLINs compared to larger households [12]. Occupation has also played a role; government employees, possibly due to better access to health information and services, have shown higher LLIN usage than farmers or daily laborers [13].

Environmental and structural factors are also critical. Seasonal variation plays a major role, as individuals tend to use LLINs primarily during the rainy season when mosquito density is perceived to be high [14]. In western Kenya, LLIN use was significantly higher in the rainy season despite similar levels of ownership year-round [15]. Housing characteristics also influence usage: households with more sleeping spaces or those with multiple rooms are more likely to use LLINs properly, while lack of space, poor ventilation, and limited net-hanging facilities reduce utilization [16].

Equally important are knowledge and perception-related factors. Households with better knowledge of malaria transmission, symptoms, and prevention are significantly more likely to use LLINs [12, 17]. Perceived susceptibility and perceived severity of malaria are often predictive of LLIN use. A study in Rwanda showed that individuals with a high perception of malaria severity were more likely to consistently use preventive measures [18]. Conversely, a study in Nigeria found that high perceived severity did not always translate into preventive behavior, particularly when confidence in net effectiveness was low [19]. This highlights the importance of self-efficacy and trust in preventive tools.

Misconceptions and cultural beliefs further complicate utilization. Some communities believe that LLINs can cause infertility, skin rashes, or even suffocation, leading to fear or avoidance [20, 21]. In areas around Lake Tana, low perceived malaria risk, beliefs in spiritual protection, and competing uses of nets such as using them for shade or as household materials, were reported [22]. Similarly, a qualitative study in East Bellessa revealed that discomfort due to heat, fear of chemicals, and limited awareness all contributed to poor LLIN usage [23].

In Gondar Zuria, despite achieving universal LLIN distribution, malaria continues to be endemic in all 44 kebeles. This district accounts for the highest malaria incidence in the Central Gondar Zone, with an incidence of 45 cases per 1,000 population in 2023, and over 234,000 people at risk. [7]. The persistence of high case numbers of points to deeper behavioral, structural and contextual barriers to LLIN utilization. These may include not only individual-level knowledge gaps and attitudes but also broader structural and environmental limitations.

Despite achieving 100% LLIN distribution in recent campaigns, Gondar Zuria District continues to report high malaria incidence, indicating a clear gap between ownership and actual use. This mismatch remains insufficiently explained in existing studies, which rarely assess the behavioral and contextual barriers that prevent proper utilization.

Given these multifaceted challenges, a purely quantitative assessment of net usage may not sufficiently capture the underlying issues. Thus, this study employed a mixed-methods approach to explore both the prevalence and determinants of LLIN utilization, while also capturing qualitative insights into the barriers households face. Through integrating these methods, the study aimed to provide a comprehensive understanding of LLIN utilization in Gondar Zuria District and to inform context-specific strategies to improve malaria prevention and move closer to national elimination goals.

## Methods

### Study design and period

A community-based cross-sectional study design was employed for the quantitative component, and descriptive qualitative study design for the qualitative component was conducted concurrently. The study was conducted from February 1 to 28, 2025.

### Study area

The study was conducted in Gondar Zuria district one of the districts in Amhara region of Central Gondar zone which is located at distance of 624 km far to Addis Ababa the city of Ethiopia to the Northwest. As of the 2024 health statistics survey report of the district, the estimated population of the district in 2025 was 245,863. From this 122,120 (49.67%) are females. Gondar Zuria district is administratively sub-divided into forty four kebeles (the lowest administrative unit). The district is located 2133 m above sea level, and it has a latitude and longitude of 12°36′N 37°28′E/12.600°N 37.467°E.

The temperature of Gondar Zuria district ranges between 16 °C and 32 °C. The average rainfall ranges from 63.3 mm ± 48.2 mm in May to 133.4 mm ± 59 mm in August. The district has 40 health posts, 8 health centers, 1 primary hospital and 13 private clinics. Malaria is endemic and its transmission peaks between September and December, and extends to summer as well,thus reflecting the climate and suitability to support mosquito breeding and malaria parasite transmission in the area.

### Source and study population

#### Source population

The study population consisted of household heads residing in the selected kebeles of Gondar Zuria District. In cases where the household head was unavailable, a responsible adult (≥ 18 years old) who could provide reliable information about the household was voluntarily interviewed as a proxy.

#### Study population

The study population is households in selected kebeles were our study population.

### Inclusion and exclusion criteria

#### Inclusion criteria

All households that have resided in Gondar Zuria for at least 6 months at the time of data collection were included in the study.

#### Exclusion criteria

Households where no responsible adult was available after two re-visits to provide information were excluded. For the qualitative part, individuals who were critically ill or mentally unable to participate in interviews at the time of data collection. Responsible adult is any household member aged 18 years or older who is mentally and physically capable of providing accurate household information.

### Sample size determination and sampling procedures

#### Sample size determination

The sample size was determined by using a single population proportion formula considering the following statistical assumptions: a 95% confidence level, the proportion of LLIN utilization 91.9% proportion from a similar previous study [24], and the marginal error of 3% (e = 0.03).

$$n=\frac{{({z}_{2 }^{\alpha })}^{2} p(1-p)}{{(d)}^{2}}$$

where n is the minimum sample size required for the study; Z is the standard normal distribution (Z = 1.96), with a confidence interval of 95% and ⍺ = 0.05; d is the absolute precision or tolerable margin of error (d) = 3% = 0.03.

$$\mathrm{n}= \frac{{(1.96)}^{2} 0.919(1-0.919)}{{(0.03)}^{2}}=317.7=318(\text{round up})$$

After adjusting design effect 2 and adding 10% non-response rate, the final sample size was 700 study participants.

The sample size for objective two is calculated using the double proportion formula, considering associated factors from previous studies [11, 24]. Among the various calculated sample sizes, the largest value of 700 was selected as the required sample size to ensure sufficient power for the study. The total sample size for qualitative data was determined by level of data saturation and homogeneous sampling was conducted to select the study participants (see Table 1).

**Table 1:** Sample size determination for factors associated with ITNs utilization

Associated factors	P1 (%)	P2 (%)	Power	CI	OR	S-1	DE	NRR	S-2
Households with pregnant women	95.4	81.4	80%	95%	4.7	192	2	10%	425
Knowledge about transmission malaria	81.5	93.2	80%	95%	0.32	286	2	10%	630

P1 = proportion of exposed individuals, P2 = proportion of unexposed individuals,S-1 = initial sample, DE = design effect, NRR = nonresponse rate, S-2 final sample

#### Sampling procedure/technique

A multi-stage sampling technique was employed to select study participants. Initially, a complete list of the 44 kebeles (clusters) within the district was compiled. Household lists were obtained from the district health office and each kebele administration. From this list, 11 kebeles representing 25% of the total were randomly selected. Following this, the total sample size was proportionally allocated to the selected kebeles based on their population size.

A systematic random sampling method was used to select households in each kebele. The sampling interval (K) was determined by dividing the total number of households in each kebele by the number of households required to be interviewed in that kebele. Using the formula, k= $\frac{N}{n}$ and taking Minziro as an example k = $\frac{1065}{52}$≈20. To ensure randomness, the first household was selected randomly using a lottery method. Once the first household is identified, subsequent households were selected systematically by adding the sampling interval (K = 20) to the position of the previously selected household through the village. This process continued until the desired sample size for the kebele is achieved. The respondents were the heads of households and where the head of the household was not available any family member whose age is above 18 years old was taken as a respondent.

**Fig. 1:**
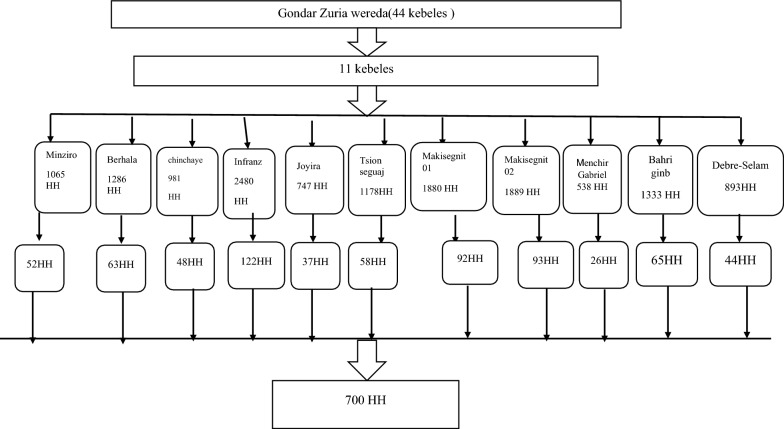
Sampling procedures, Gondar Zuria district February 2025

Purposive sampling was employed to select participants for the qualitative component of the study. A total of 10 participants were recruited for the qualitative component, with recruitment ceasing upon reaching data saturation, the point at which no new themes or information emerged from subsequent interviews. Key informants were purposively selected based on their direct involvement in malaria prevention activities, familiarity with local LLIN distribution and usage practices, and their roles as health extension workers, malaria focal persons, and community leaders. Data saturation was monitored continuously throughout data collection and was considered achieved when no new themes, codes, or insights emerged from subsequent interviews. Saturation was reached after seven in-depth interviews and three key informant interviews (see Fig. 1).

### Variables of the study

#### Dependent variable

The dependent variable of this study was LLIN utilization, categorized as “yes” if all the household members slept under a long-lasting insecticide-treated net the previous night and “no” otherwise.

#### Independent variables

Independent variables included sociodemographic characteristics such as age, sex, educational status, marital status, occupation, and household size, as well as malaria-related factors such as knowledge about malaria transmission and prevention, perceived susceptibility and severity, previous malaria history, and distance to the nearest health facility.

### Operational definitions

**Household head:** any person either male or female who owns a particular house and decides for the entire family [16].

**Utilization of bed net**: if the respondent reported that all members of the household slept under the bed net in the night prior to the day of data collection and supported with an observation of a hanged net over the sleeping beds or areas [16].

**Perceived susceptibility** refers to the perception of a respondent about the chance of getting a malaria infection and was assessed through 4 items of questions [23, 26]. It was classified as high perceived susceptibility (i.e., a score of ≥ the mean score) and low perceived susceptibility (i.e., a score of < the mean value).

**Perceived severity** refers to the beliefs of the respondents about the seriousness or severity of the malarial disease and was assessed through 6 items of questions [23, 26]. It was classified as high perceived severity (i.e., a score of ≥ the mean value) and low perceived severity (i.e., a score of < the mean value).

**Self-efficacy** refers to the perception or confidence of respondents towards ITN utilization and was assessed through 3 items of questions [23, 26]. It was reported as high perceived self-efficacy (i.e., a score of ≥ the mean value) and low perceived self-efficacy (i.e., a score of < the mean value.

**Knowledge about malaria** was measured by using 6 items of knowledge assessing questions related to causes, transmission and prevention methods of malaria. Each correct answer was given a score of 1, while incorrect or "don’t know" responses were scored as 0, resulting in a total possible score ranging from 0 to 6.Based on Bloom’s cutoff point, knowledge levels were categorized as follows: Those who correctly answered 80% or more of the questions were classified as having high knowledge, between 60 and 79% were considered to have moderate knowledge, while those with less than 60% correct answers were considered to have low knowledge[21, 22].

**Knowledge about LLINs** was measured by using 6 items of knowledge assessing questions related to LLIN use, benefits, and malaria prevention. Each correct answer was given a score of 1, while incorrect or "don’t know" responses were scored as 0, resulting in a total possible score ranging from 0 to 6.Based on Bloom’s cutoff point, knowledge levels were categorized as follows: Those who correctly answered 80% or more of the questions were considered as having high knowledge,individuals who scored 60% to 79% were classified as having moderate knowledge, while those with correct answers below 60% were considered to have poor knowledge[21, 22].

**LLIN-to-family size ratio**: This ratio was calculated by dividing the total number of Long-Lasting Insecticidal Nets (LLINs) available in a household by the total number of household members. According to the WHO recommendation for universal coverage, one LLIN should be available for every two individuals (i.e., a ratio of 0.5). Based on this standard, the ratio was classified as:

**Low coverage**: Ratio < 0.5, indicating insufficient LLINs to cover all household members.

**Adequate coverage**: Ratio ≥ 0.5, indicating sufficient LLINs, with at least one net available for every two individuals in the household.

**Responsible adult**: Any household member aged 18 years or older who is mentally and physically capable of providing accurate household information [32].

### Data collection tool and procedure

The data was collected using quantitative and qualitative data collection techniques. Both quantitative and qualitative data were collected concurrently but separately**,** using different samples and tools. The two datasets were later integrated to allow for triangulation and complementarity in interpretation. For quantitative part, data was collected using a structured questionnaire adapted from different literatures was administered using paper-based methods initially developed in English and translated into Amharic. The collection process combined face-to-face interviews with direct observations. To verify LLIN usage, enumerators cross-check interview responses with physical observations, ensuring that LLINs are properly hung above sleeping areas. A supervisor, who is malaria prevention and control program officers from the district health office, along with the principal investigator, controlled the data collection process and ensured quality control. The data was collected by BSc graduate health professionals. Key informant interviews were employed as the primary method for qualitative data collection. A semi-structured interview guide was used for participants to elaborate on their responses. The key informants’ interviews were employed for about 10 to 20 min for each respondent until the point of saturation was reached. The interviews were recorded using a recorder with the participants' verbal consent, and notes were taken during the process.

### Data quality control

For quantitative data quality control, a pre-test was done on 5% of sample participants in the East Dembia district. A total of 2 BSc nurses were trained for one day in data collection and handling. Closed supervision by the principal investigator was done until the data collection time ended. The qualitative data was collected by the principal investigator and Master of Public Health graduate.

### Data processing and analysis

The data was entered into Epi-data version 4.7 data entry software and then exported to SPSS version 25 for cleaning, coding, and analysis. Descriptive statistics were computed and reported using text, tables, and graphs. A binary logistic regression model was fitted to identify factors associated with LLINs utilization.Variables with a p ≤ 0.25 in the binary logistic regression were considered candidates and entered into the multivariable logistic regression model to control for potential confounding. Multicollinearity was checked using the variance inflated factor (mean VIF = 1.04). The Hosmer and Lemeshow goodness of fit test was computed.

An adjusted odds ratio with a 95% confidence interval was computed to see the presence, strength, and direction of association between the dependent and independent variables. g and identify independent predictors of LLIN utilization. Finally, variables with a p-value of < 0.05 at 95% CI with their AOR were considered to have a statistically significant association with LLINs utilization.

Qualitative data were recorded, and interviews were transcribed verbatim, and the transcribed data was also translated to English by the principal investigator. Transcripts were analyzed using the principles of thematic analysis in Open Code 4.02 software. Initial coding was conducted within each transcript to identify key concepts and patterns. To integrate the quantitative and qualitative strands, a triangulation approach was used during interpretation. After identifying significant quantitative associations, qualitative themes were examined to provide explanatory context for these patterns, particularly regarding misconceptions, seasonal usage behavior, and structural barriers. This integration allowed the qualitative findings to clarify why certain quantitative relationships emerged and supported the development of more holistic and context-specific conclusions.

### Ethical consideration

The study was conducted in full compliance with ethical standards. Ethical clearance was obtained from the Institutional Review Board (IRB) of the University of Gondar, College of Medicine and Health Sciences, Institute of Public Health Ethical Review Board (ref No/IPH/3157/2025). Following this, support letters were secured from the Regional Health Bureau, Zonal Health Department, and the selected district health office. The selected district issued supportive letters to their respective health centers to facilitate data collection.

Before commencing data collection, written informed consent was obtained from each study participant. The consent process involved a clear explanation of the study's purpose, procedures, potential risks, and benefits. Throughout the study, privacy and confidentiality was strictly maintained. Data is anonymized and used solely for the purposes of this research. The principles of justice, respect, and beneficence was upheld to ensure the ethical treatment of all participants during the entire research process.

## Results

### Socio-demographic characteristics of the respondents

A total of 700 respondents participated in the quantitative study resulting in a response rate of 100%. About 383(54.7%) of the respondents were males. More than half (58.9%) of the participants were in the age group of 26–45-year-old with the mean ± standard deviation (SD) of 36.5 ± 12.8. Regarding marital status, 474 (67.7%) of respondents were married, whereas 197(28.1%) were single. In terms of religious affiliation, the majority were Orthodox Christian 602(86%). About 273(39%) of respondents could read and write. Regarding Occupation, 271(38.7%) of respondents were farmers, and 90(12.9%) were government employees. Family size distribution showed that 278(39.7%) of households had 4–5 members (Table 2).

**Table 2:** Socio demographic and household characteristics of the respondents in Gondar Zuria district, northwest Ethiopia, 2025

Variables		Frequency	%
Sex	Male	383	54.7
	Female	317	45.3
Age of respondents	18–25	133	19
	26–45	412	58.9
	> 45	155	22.1
Marital status	Single	197	28.1
	Married	474	67.7
	Divorced	19	2.7
	Widowed	10	1.4
Religion	Orthodox	602	86
	Muslim	98	14
Educational status	Cannot read and write	171	24.4
	Can read and write	273	39
	Primary education	106	15.1
	Secondary education	57	8.1
	College and above	93	13.3
Occupation	Farmer	271	38.7
	Housewife	125	17.9
	Government employee	90	12.9
	Merchant	130	18.6
	Daily labourer	14	2.0
	Others	19	2.7
	Jobless	51	7.2
Roof of the house	Corrugated iron	664	94.9
	Thatched roof	36	5.1
Total number of beds/sleeping spaces	1	282	40.3
	≥ 2	418	59.7
Family size	1–3	218	31.1
	4–5	278	39.7
	≥ 6	204	29.1

Others = students

### Knowledge about Malaria and LLINs

Of the total number of respondents, 422 (60.3%) had a low level of knowledge regarding malaria. The majority of participants (608; 86.9%) correctly identified mosquito bites as the cause of malaria. Regarding knowledge of malaria symptoms, 214 (30.6%) recognized chills (shivering)**,** 392 (56.0%) identified fever**,** and 86 (12.3%) mentioned headache as common symptoms. Regarding awareness of malaria-vulnerable groups, 449 (64.1%) respondents identified under-five children as being at high risk, followed by 80 (11.4%) who cited pregnant women, and 37 (5.3%) who mentioned the elderly as particularly vulnerable to malaria.

Concerning the purpose of long-lasting insecticide-treated nets (LLINs), 614 (87.7%) respondents stated that LLINs are primarily used to protect against *Anopheles* mosquitoes, while the remaining participants believed they also serve other purposes. With respect to the seasonality of LLIN use, 436 (62.2%) reported using them every night throughout the year, 179 (25.6%) used them mainly during the rainy season, and the rest reported usage only when mosquito density is high.

### Perception about Malaria and LLINs

About 411(58.7%) of the respondents had high perceived susceptibility with overall mean perceived susceptibility score of 23.82 (SD = 3.27). While the overall perceived susceptibility was high, notable variations were observed across different demographic subgroups, particularly by sex. Among the 383 male respondents, 256 (66.8%) reported high perceived susceptibility. Conversely, among the 317 female respondents, 155 (48.9%) reported high perceived susceptibility.

Approximately 368 (52.6%) of the respondents reported high perceived severity of malaria. The overall mean perceived severity score was 25.66 (SD = 3.503). This varied notably across different age groups. Respondents aged 46 and above showed the highest proportion of high perceived severity, with 102 out of 155 (65.8%) individuals in this group reporting high severity. Conversely, the 25–45 age group had the lowest proportion of high perceived severity (197 out of 412, or 47.8%).

About 378 (54%) of the participants had high confidence in LLINs utilization with the mean (± SD) self-efficacy score of 23.19 (± 3.527) (see Table 3).

**Table 3:** Perception about malaria and LLINs among respondents in Gondar Zuria, Northwest Ethiopia, 2025

Variables	low	High	Total	Mean ± SD
Perceived susceptibility	289(41.3%)	411(58.7%)	700(100%)	23.82 ± 3.270)
Perceived severity	332(47.4%)	368 (52.6%)	700(100%)	25.66(± 3.503)
Self-efficacy	322(46.0%)	378 (54%)	700(100%)	23.19 (± 3.527)

### LLINs utilization

The prevalence of LLINs utilization was 55.6% (95% CI: 51.9%–59.3%). About 96 (13.7%) of the pregnant women and 251 (35.9%) of the under five children utilized LLINs. About 296 (42.3%), 250 (35.7%), 116(16.6%) and 38(5.4%) of the households had only one, only two, only three and ≥ four LLINs, respectively. Half (49.1%) of the households indicated that their LLINs were old, despite being intact without visible holes. Approximately 311 participants (44.4%) reported that all family members did not sleep under LLIN on the night preceding the survey. The most frequently mentioned reasons for not using LLINs regularly was lack of sufficient LLINs for all household members 268 (38.3%) and the perception that LLINs cause excessive heat 37 (5.3%).The mean number of LLINs per household was 1.8, ranging from 1 to 4.

### Factors affecting LLINs utilization

Following multivariable analysis, three variables namely perceived severity**,** LLINs to family size ratio and occupation were found to be significantly associated with LLIN utilization at a p-value ≤ 0.05. Occupation (government employees) showed 5 times higher odds of LLIN use compared to farmer (AOR = 4.86; 95%CI 2.62–9.04). Additionally, higher perceived severity was associated with increased LLIN utilization(AOR = 1.50;95%CI 1.08,2.07).Finally, households possessing an adequate ratio of LLINs to family size were found to have 2.09 times higher odds of LLINs utilization compared to households with an inadequate ratio.(AOR = 2.09; 95% CI 1.51,2.91) (see Table 4).

**Table 4:** Factors associated with ITN utilization among households, Gondar Zuria District, northwest Ethiopia, 2025

Variables	Categories	LLINs utilization		COR(95%CI)	P-value	AOR(95%CI)	P-value
		Yes	No				
Occupation	Farmer	131	147	1		1	
	Housewife	58	69	0.94(0.61,1.43)	0.78	0.84(0.54,1.31)	0.45
	GE^*^	75	15	5.61(3.07,10.24)	0.001	**5.07(2.73,9.42)**	0.001^*^
	Merchant	82	53	1.73(1.14,2.63)	0.01	1.46(0.94,2.26)	0.08
	DL^*^	11	5	2.46(0.83,7.29)	0.10	2.59(0.85,7.90)	0.29
	Jobless	32	22	1.63(0.90,2.94)	0.15	1.37(0.74,2.51)	0.31
Perceived susceptibility	Low	176(60.8%)	113(39.1%)	1		1	
	high	213(51.8%)	198(48.2%)	0.83(0.61,1.13)	0.24	0.93(0.67,1.28)	0.65
Perceived severity	Low	160(48.2%)	172(51.8%)	1		**1**	
	high	229(62.2%)	139(37.8%)	1.77(1.31,2.39)	0.001	**1.50(1.08,2.07)**	0.01^*^
Self-efficacy	Low	165(51.2%)	157(48.8%)	1		1	
	High	224(59.3%)	154(40.7%)	1.38(1.02,1.86)	0.03	1.08(0.78,1.49)	0.63
LLINs/family size ratio	Inadequate	187(47%)	211(53%)	1		1	
	adequate	202(66.9%)	100(33.1%)	2.27(1.67,3.10)	0.001	**2.09(1.51,2.91)**	0.001^*^

GE^*^ = government employee,DL^*^ = daily laborer||*p_value ≤ 0.05

### Qualitative findings on barriers to LLINs utilization

For qualitative part, 7 in-depth interviews and 3 key informants were included. About 7(70%) were males and 9 of them were in the age group 26–45. About 4(40%) didn’t attend formal education and 8(80%) were married. The qualitative interviews explored perceptions, beliefs, experiences, and practices related to Long-Lasting Insecticide-Treated Nets (LLINs) among households. Three themes were identified through thematic analysis: perceptions and beliefs about LLINs, practical challenges to consistent LLINs use and alternative malaria prevention and non-health use of LLINs. Each theme is further elaborated with sub-themes and supported by illustrative quotes.

#### Perceptions and beliefs about LLINs

Participants demonstrated varying levels of understanding and beliefs regarding LLINs. While some appreciated their role in malaria prevention, others expressed uncertainties or made misconceptions.

#### Misconceptions about LLINs

Some respondents believed that LLINs could cause health issues such as infertility or skin irritation due to the insecticide, discouraging consistent use. Other participants explained that they feared the chemicals on the LLINs could weaken the body or cause breathing difficulties at night, and this perception discouraged them from using the nets consistently. *"Using the net for a long time can make women infertile." (a 45-year old male farmer at Bahri ginb kebele).* A few believed that the chemical used in the nets was harmful to health, leading them to avoid using them. *"The smell of the net is bad, and I think it can harm our health, so we don’t use it often." (a 29 year old female daily laborer in Bahri ginb kebele).*

#### Doubts about LLINs effectiveness

Several participants questioned the effectiveness of LLINs, particularly when malaria cases occur despite net usage or when the nets are old or damaged. *"We sleep under it, but sometimes mosquitoes still bite us." (A 31 year old male farmer in Infranz kebele)*.

#### Practical challenges to consistent LLINs Use

Multiple structural and contextual factors affected the regular and proper use of LLINs.

#### Household environmental constraints

Participants mentioned limitations such as lack of space, especially in crowded or poorly ventilated homes, making it difficult to hang nets. *“There is not enough room to hang the net in our house." (a 36 year old housewife in Makisegnit kebele 01).* Many respondents noted that the nets deteriorated quickly, becoming less protective and leading to reduced motivation for use. *“Our net is torn, and we get bitten even when we use it." (50 year old farmer in Bahri ginb kebele).*

#### Seasonal usage patterns

LLIN usage was found to be seasonal, as the majority of households used them only during the rainy season or shortly after, when mosquito density is believed to be high. During dry season, nets were found to be folded and stored, or even discarded, on the premise that risk of malaria transmission was low. Participants also mentioned discomfort brought about by heat as a primary hindrance to use during hot weather. This seasonal pattern of use suggests that consistent year-round protection is lacking, potentially reducing the overall effectiveness of LLINs in malaria prevention *“We use them only in the rainy months when the mosquitoes are many." (a 40 year old female farmer in Infranz kebele). Another participant described that during the dry season, the heat under the net becomes unbearable, creating a sensation ‘like sleeping inside a closed plastic bag,’ which discourages nightly use. (A 31 year old male farmer in Infranz kebele).*

#### Alternative Malaria prevention and non-health use of LLINs

Some participants reported repurposing LLINs or relying on alternative prevention strategies.

#### Alternative prevention methods

Indoor residual spraying (IRS) and the use of antimalarial medication were sometimes used in place of LLINs by some households. Some of the interviewees believed that spraying was more immediate and longer term in keeping mosquitoes away, especially where the nets were worn or perceived as less effective. Some other interviewees wanted to take medication after falling sick instead of using bed nets as a preventive method on a regular basis. Such preference was typically associated with discomfort concerning the use of LLINs, such as heat. As a result, others saw LLINs as the second option rather than the first preventive. *"Spraying is easier; you don’t have to deal with the heat inside the net. I sometimes choose to be treated with medication if malaria contracts me, rather than consistently using bed nets.* "(*A 31 year old male farmer in Infranz kebele*).

#### Repurposing LLINs for none-health uses

Some of the families reported that they re-purposed LLINs for non-health purposes, such as shade seedlings, to use as a grass-carrying device, or for making rope. This was normally based on the perception that the LLINs devalue after a short time and are unable to achieve the target of three years of usage. Some of the participants doubted that the nets were durable and believed that they would be useful for only a short time after distribution. Thus, when the nets were considered "old" or "not usable," they were diverted towards alternative purposes. *"We use the old ones to tie plants or carry grass." (41 year old woman merchant in Infranz kebele).*

## Discussion

This study assessed the utilization and associated factors of LLINs among households in Gondar Zuria District. The findings revealed that LLIN utilization was suboptimal, with only slightly more than half of the respondents reporting use the night before the survey. Both individual-level perceptions and contextual barriers were found to influence net use.

In this study, the utilization of LLINs was found to be 55.6% (95% CI: 51.9%–59.3%). which is comparable to findings from east Bellessa,(56.5%) [23], East Meskan in Gurage zone (58.2%) [27], Itang in Gambella region (52.3%) [18]. This indicates a consistent pattern of moderate LLIN utilization across various settings in the country, reflecting similar challenges in consistent usage or behavioral factors. However, the utilization rate observed in this study is lower than that reported in Kola Diba Town (91.1%) [10], Kersa in Jimma zone(83.5%) [11], Ilu Galan in Oromia region (72.2%) [17], Mirab Abaya District in Southern Ethiopia(85.1%) [28].

This variation may be partly due to differences in how LLIN utilization was measured. In the present study, utilization was defined as all family members sleeping under a bed net, and data were collected through direct observation of bed nets hanging over sleeping spaces. In contrast, other studies considered LLIN utilization if at least one household member reported using a bed net the night before data collection [28]. Another reason for the discrepancy could be the study population: most previous studies focused on high-risk groups such as children under five and pregnant women, whereas this study assessed utilization at the household level.

In addition, this study combined direct observation with self-reported information to assess LLIN utilization, which enhanced the objectivity of the data and minimized the potential influence of social desirability bias typically seen in self-reported responses. Furthermore, urban or peri-urban settings like Kola Diba Town might benefit from better access to health information, or different housing structures that facilitate easier net hanging, leading to higher rates. In contrast, rural or remote areas like Gondar Zuria might face greater challenges related to dispersed populations or reduced access to routine health education. This challenge was expressed by a 36-year-old female participant: *"We need health education, but the health extension workers do not come to visit us recently like they used to because of the security issues*.". Follow-up mechanisms, or the duration since the last mass distribution campaign could also play a significant role. One participant emphasized the absence of post-distribution monitoring, stating, *“There’s also a lack of follow-up to ensure the nets are being used properly.” (35-year-old male resident).* This shows that there is a potential gap in household-level follow-up, and which lead to poor usage or misuse of LLINs over time. Such gaps may contribute to the observed discrepancy between net ownership and effective utilization in the study area.

The multivariable logistic regression analysis identified three significant factors associated with LLIN utilization: occupation (specifically being a government employee), high perceived severity of malaria, and an adequate LLIN-to-family size ratio. Perceived susceptibility and beliefs that LLINs contain harmful chemicals were not statistically significant in the final model and are therefore not discussed as independent predictors.

In this study occupation showed a statistically significant association with LLINs utilization. Accordingly, government employees were more likely to use LLINs 3.75 times more compared to jobless individuals. This finding is consistent with previous studies, which suggest that individuals who are employed may have improved access to health information and preventive tools, including LLINs [16]. Government employees may also be more likely to participate in health campaigns or have better awareness of malaria prevention practices due to their education level or occupational exposure to public health information.

Perceived severity of malaria was another significant factor. Households with a high perceived severity of malaria were 1.52 times more likely to use LLINs compared to those who perceived malaria as less severe. This finding agrees with a study conducted in Rwanda [25]. But this finding contradicts with a study conducted in Nigeria which claims that counterintuitively, those perceiving higher severity had a slightly lower likelihood of using LLINs [29]. This suggests that without accompanying beliefs in the efficacy of prevention methods, heightened perceived severity alone may not motivate protective behaviors and perceived severity does not always translate to preventive action.

Households with an adequate number of LLINs relative to their family size were 2.09 times more likely to use them compared to those with an inadequate LLINs. When the number of nets is insufficient, households may prioritize vulnerable groups such as young children and pregnant women, while leaving other members unprotected. This finding agrees with a study conducted in Kenya [30]. In many households, LLINs are not used simultaneously by all family members, even though distribution was based on the standard one net per two individuals. This may be due to practical challenges such as limited sleeping space or insufficient areas to hang the nets properly, which reduces the net-to-person ratio and undermines the intended level of utilization. This finding is also supported by an interview claiming like *"The bed net is old, and we do not have a new one. We used them all that the government gave us two years ago. Why don’t they give us a new one?"-36 year old female respondent.*

In addition, the physical structure of many households in Gondar Zuria, characterized by small, overcrowded rooms with poor ventilation limits the ability to hang nets properly and makes sleeping under a net uncomfortable, especially during the dry season. The district has also faced intermittent security challenges in recent years, reducing the frequency of household visits and community-level engagement by health workers. Participants also pointed to heat discomfort as a major deterrent during dry seasons, leading to seasonal usage patterns. This is also explained by an interview claiming, *"during hot weather, the bed net traps heat, making it uncomfortable to sleep under.”* Seasonal usage primarily during rainy months has been documented elsewhere in Ethiopia and West Africa, often due to the perceived link between mosquito density and rainfall [31]. However, this practice may leave households vulnerable to malaria transmission during dry seasons and protracted outbreaks. These contextual and behavioral barriers help explain why high LLIN ownership in Gondar Zuria has not translated into higher utilization rates and highlight the need for targeted, locally sensitive malaria prevention strategies.

Based on these findings, several targeted interventions are recommended to improve LLIN utilization in Gondar Zuria District. Strengthening regular community follow-ups by health extension workers is essential to reinforce correct LLIN usage, address misconceptions, and monitor net conditions at the household level. Targeted educational campaigns using locally accessible platforms such as community gatherings, religious institutions, schools, and local media should prioritize correcting misinformation, promoting year-round use, and explaining the continued importance of LLINs even in dry seasons. In addition, establishing simple community-based LLIN monitoring systems that track damaged nets and ensure timely replacements would help maintain functional coverage. Integrating LLIN promotion with existing community structures, such as the Health Development Army, can further support sustained behavioral change and contribute to stronger malaria control efforts in the region.

Despite this study provides meaningful findings on LLINs utilizations, there are some limitations that should be considered while interpretation the findings. Data was collected during a dry season, which may not fully capture seasonal variations in LLIN utilization. LLIN use often fluctuates with perceived high mosquito density, which tends to increase during peak transmission seasons though this limitation is corroborated by the qualitative findings.

## Conclusion

LLINs utilization (55.6%) in Gondar Zuria District was below the national target for malaria prevention (100%). Utilization was significantly higher among government employees, high perceived severity of malaria and high LLINs to family size ratio. Our findings also revealed that misconceptions and beliefs have significantly hindered the consistent and year-round use of LLINs. Many participants doubted the effectiveness of the nets, especially when they are getting old. Such perceptions have contributed to irregular usage patterns and reduced trust in LLINs as a reliable malaria prevention method.

Despite universal LLIN distribution in Gondar Zuria District, utilization remains far below expected levels due to persistent misconceptions, doubts about LLIN effectiveness, household structural limitations, and strong seasonal patterns in net use. Addressing these barriers requires targeted and continuous health education that corrects misconceptions and promotes the importance of using nets throughout the year. Regular household-level follow-ups by health extension workers, combined with community-based forums such as schools, religious institutions, and neighborhood groups, can reinforce proper usage practices and support long-term behavior change. Establishing a simple system for monitoring net condition and ensuring timely replacement of damaged LLINs will also be crucial for maintaining effective coverage. Ensure household-level net allocation is calculated to truly meet the WHO minimum ratio of 1 net per 2 people. Strengthening these actionable strategies is essential for improving LLIN utilization in Gondar Zuria District and contributing to Ethiopia’s broader malaria elimination goals.

## Data Availability

The datasets generated and/or analyzed during the current study are not publicly available due to participant confidentiality but are available from the corresponding author on reasonable request.

## Abbreviations

AOR: Adjusted odd ratio
BCC: Behavior change communication
CI: Confidence interval
DE: Design effect
DL: Daily laborer
GE: Government employee
HH: Household
IRS: Indoor residual spraying
ITNs: Insecticide-treated nets
LLINs: Long-lasting insecticide-treated nets
